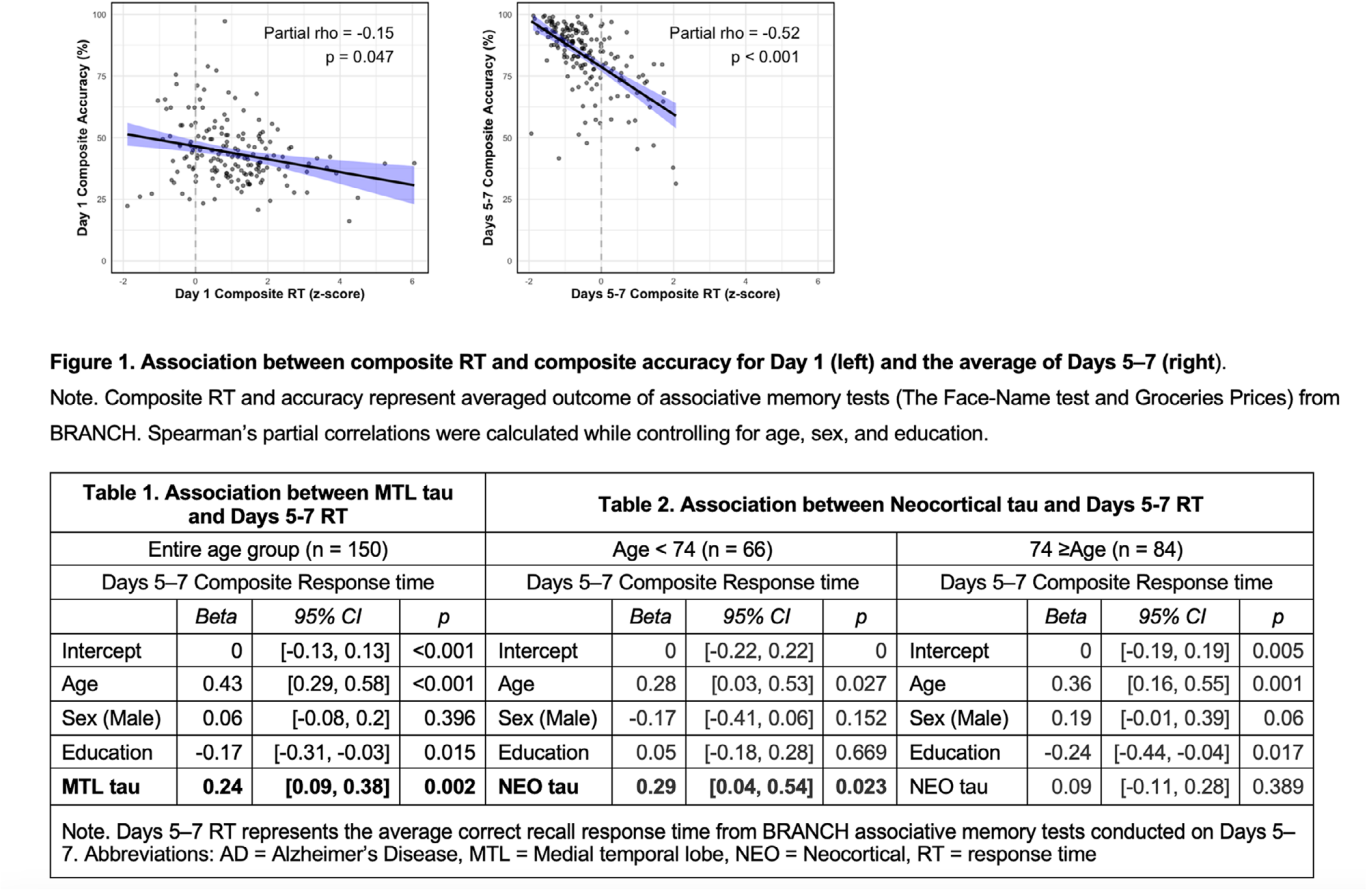# Utilizing Memory Recall Response Time on Digital Cognitive Testing to Detect Subtle Cognitive Changes Associated with Preclinical Alzheimer's Disease

**DOI:** 10.1002/alz70857_104903

**Published:** 2025-12-25

**Authors:** Hairin Kim, Roos J Jutten, Daniel Soberanes, Mark A. Dubbelman, Lia D'Aquila, Onyinye Udeogu, Gad A. Marshall, Keith A. Johnson, Dorene M. Rentz, Reisa A. Sperling, Kathryn V Papp, Rebecca E. Amariglio

**Affiliations:** ^1^ Brigham and Women's Hospital, Harvard Medical School, Boston, MA, USA; ^2^ Alzheimer Center Amsterdam, Neurology, Vrije Universiteit Amsterdam, Amsterdam UMC location VUmc, Amsterdam, Netherlands; ^3^ Massachusetts General Hospital, Harvard Medical School, Boston, MA, USA; ^4^ Center for Alzheimer's Research and Treatment, Department of Neurology, Brigham and Women's Hospital, Harvard Medical School, Boston, MA, USA; ^5^ Center for Alzheimer Research and Treatment, Department of Neurology, Brigham and Women's Hospital, Boston, MA, USA; ^6^ Center for Alzheimer's Research and Treatment, Brigham and Women's Hospital, Harvard Medical School, Boston, MA, USA

## Abstract

**Background:**

Response time (RT), traditionally linked to age‐related processing speed, may also reflect memory consolidation—stabilizing and reorganizing information for faster retrieval. Alzheimer's disease (AD) pathology, particularly in hippocampal‐cortical networks, disrupts consolidation, potentially prolonging retrieval latency before overt memory decline. We examined whether memory recall RT for correct trials evolves with repeated learning and correlates with amyloid and tau burden in the brain.

**Method:**

A total of 175 cognitively unimpaired older adults (Age=74.21±8.33, Education = 16.55±2.50, Female=66.9%) completed seven days of daily associative memory tests with the same stimuli each day using the Boston Remote Assessment for Neurocognitive Health (BRANCH). Because memory consolidation is expected to strengthen over repeated exposures, we considered Day 1 a “early‐consolidation” phase and Days 5–7 a “late‐consolidation” phase. Then, we compared RT‐accuracy correlation across these phases. In a PET‐imaged subsample (*n* = 150), late‐consolidation RT (averaged RT across Days 5–7) was regressed on cortical amyloid, medial temporal tau, and neocortical tau, adjusting for age, sex, and education. Given age's known impact on processing speed, a Johnson–Neyman analysis was utilized to identify a potential age‐specific association between RT and AD‐related pathology.

**Result:**

Recall RT shortened significantly from the early‐consolidation phase (Day1: M=3.52s, SD=1.26) to the late‐consolidation phase (Days 5–7: M=2.68s, SD=0.66), reflecting improved recall efficiency. The RT‐accuracy correlation strengthened in the late‐consolidation phase (Day1: ρ=‐0.15, *p* = 0.047, Days 5–7: ρ=‐0.52, *p* <0.001), supporting that RT reflects memory consolidation. Slower late‐consolidation RT correlated with higher medial temporal tau (β=0.24, *p* = 0.002) but not with neocortical tau (β=0.12, *p* = 0.137) and amyloid (β=‐0.11, *p* = 0.527). An age‐specific relationship emerged only for neocortical tau and late‐consolidation RT among individuals <74 (β=0.29, *p* = 0.023), whereas RT among those ≥74 was explained solely by age (β=0.36, *p* <0.001).

**Conclusion:**

Even among correct responses, recall speed—a novel and underexplored metric—revealed critical insights: RT tracks memory consolidation and functions as a pathology‐specific marker, complementing traditional accuracy measures susceptible to ceiling effects in individuals without overt cognitive decline. Furthermore, we found that tau burden concurrently affects RT alongside age effect, with pronounced effects in individuals under 74—highlighting its potential to detect early Alzheimer's disease pathology.